# Alternative Splicing: A Potential Therapeutic Target in Hematological Malignancies

**DOI:** 10.3390/hematolrep16040066

**Published:** 2024-10-29

**Authors:** Gazmend Temaj, Silvia Chichiarelli, Sarmistha Saha, Pelin Telkoparan-Akillilar, Nexhibe Nuhii, Rifat Hadziselimovic, Luciano Saso

**Affiliations:** 1 Faculty of Pharmacy, College UBT, 10000 Prishtina, Kosovo; gazmend.temaj@ubt-uni.net; 2 Department of Biochemical Sciences “A. Rossi-Fanelli”, Sapienza University of Rome, 00185 Rome, Italy; silvia.chichiarelli@uniroma1.it; 3 Department of Biotechnology, Institute of Applied Sciences & Humanities, GLA University, Mathura 00185, Uttar Pradesh, India; 4 Department of Medical Biology, Faculty of Medicine, Gazi University, 06500 Ankara, Turkey; pelintelkoparanakillilar@gazi.edu.tr; 5 Department of Pharmacy, Faculty of Medical Sciences, State University of Tetovo, 1200 Tetovo, North Macedonia; nexhibe.nuhii@unite.edu.mk; 6 Faculty of Science, University of Sarajevo, 71000 Sarajevo, Bosnia and Herzegovina; rifat.hadziselimovic@gmx.net; 7 Department of Physiology and Pharmacology “Vittorio Erspamer”, La Sapienza University, 00185 Rome, Italy; luciano.saso@uniroma1.it

**Keywords:** hematologic cancers, alternative splicing factor, RNA splicing, therapeutics

## Abstract

Leukemia represents the most prevalent malignancy in children, constituting 30% of childhood cancer cases, with acute lymphoblastic leukemia (ALL) being particularly heterogeneous. This paper explores the role of alternative splicing in leukemia, highlighting its significance in cancer development and progression. Aberrant splicing is often driven by mutations in splicing-factor genes, which can lead to the production of variant proteins that contribute to oncogenesis. The spliceosome, a complex of small nuclear RNAs and proteins, facilitates RNA splicing, a process critical for generating diverse mRNA and protein products from single genes. Mutations in splicing factors, such as U2AF1, SF3B1, SRSF2, ZRSR2, and HNRNPH1, are frequently observed across various hematological malignancies and are associated with poor prognosis and treatment resistance. This research underscores the necessity of understanding the mechanisms of RNA splicing dysregulation in order to develop targeted therapies to correct these aberrant processes, thereby improving outcomes for patients with leukemia and related disorders.

## 1. Introduction

Leukemia is the most frequent malignancy disorder, accounting for 30% of childhood cancer diagnoses [1]. All occur at a rate of approximately 186.6 cases per 1 million individuals, with the vast majority, about 95%, diagnosed in childhood. Acute lymphoblastic leukemia (ALL) is known as a heterogeneous disease. Alternative splicing plays a crucial role in the development of many cancers and is in particular a distinctive feature of leukemias with mutations in genes encoding splicing factors (SF) [2]. Aberrations in gene alternative splicing can stem from various factors that are involved in this process. These include alterations in the expression of key regulatory alternative splicing factors, such as proto-oncogenes or tumor suppressor genes. These changes can lead to the production of variant proteins that may contribute to cancer initiation, progression, and metastasis [3,4,5]. Understanding these mechanisms is essential for developing targeted therapies to combat cancer. Many molecular inhibitors and compounds have been developed as therapeutics, which have been shown to be involved in preventing the splicing factors of oncogenes [6]. Dysregulation of RNA splicing commonly plays a role in cancer cells by disrupting molecular processes known as “cancer hallmarks” [6].

RNA splicing involves the removal of non-coding sequences from pre-mRNA to form mature mRNA. This dynamic process is carried out by the spliceosome complex. The spliceosome complex consists of large ribonucleoprotein complexes composed of small nuclear RNAs (snRNAs) and proteins known as splicing factors. In eukaryotic cells, the spliceosome complex is divided into two types: the major and minor spliceosomes. The majority of introns, approximately 99.5%, which contain the GT-AG sequence at the 5′ splice site, are recognized and removed by the major spliceosome [7]. The minor spliceosome (“U12-dependent introns”) operates in 0.5% of human genes and is defined by conserved 5′ and 3′ oligonucleotides [7,8,9,10]. 

Mutations in splicing factors are common in acute myeloid leukemia (AML) and myeloproliferative neoplasms (MPN) [11,12,13,14,15,16,17]. More than 50% of patients diagnosed with myelodysplastic syndromes (MDS) carry mutations in RNA splicing-factor genes [11,12,13,18]. The splicing-factor mutations and their effect on RNA are described by many other authors [19,20,21,22,23]. 

## 2. Regulation of the RNA Splicing

RNA splicing plays a pivotal role in regulating gene expression, enabling a single gene to produce multiple proteins. The spliceosome machinery, consisting of five small nuclear ribonucleoproteins (snRNPs; U1, U2, U4, U5, and U6 snRNP), catalyzes this process. Each of these molecules contains its own small nuclear RNA (snRNA) complexed with a protein, with over 200 proteins in total [24]. The sequence embedded in the pre-mRNA can base pair with snRNAs and recruit the spliceosome at the 5′ splice site (located in the 5′ end of the sequence to be removed), the 3′ splicing site (located in the 3′ end of the removed sequence), the branch point sequence (BPS, located upstream of the 3′ splice site), and the polypyrimidine tract, which is located between the BPS and the 3′ splicing site. Spliceosome assembly is facilitated by U1 snRNP, which connects to the 5′ splicing site; SF1 (splicing factor 1), which binds to the BPS; and the auxiliary factor U2AF, which connects to the polypyrimidine tract at the 3′ splice site. The recognition of splice sites by the spliceosome is enhanced by additional auxiliary splicing factors that facilitate the recruitment of the spliceosome [25]. These auxiliary factors include members of the SR (serine/arginine) family, which typically aid splicing by recognizing specific splicing sequences in the pre-mRNA (both intron and exon sequences). On the other hand, heterogeneous nuclear ribonucleoproteins (hnRNPs) typically suppress the splicing process by interacting with intron and exon splicing silencers [26]. The main enzymatic steps involved in RNA splicing are the two sequential transesterification reactions. The nucleotide positioned at the branch-point site initiates a nucleophilic attack, facilitating lariat formation.

**Figure 1:**
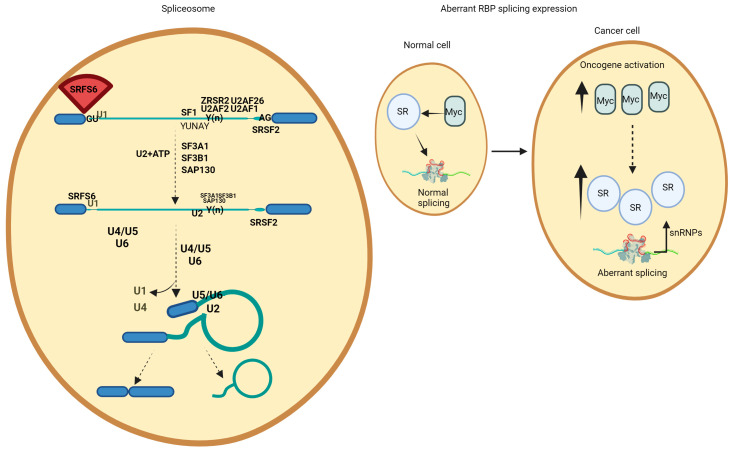
The main steps of mRNA splicing. The steps of this process are catalyzed by spliceosomes and specified by an RNA sequence: the 5′ splice site, the 3′ splice site, and the branch site (yUnAy). The splicing site 5′ is recognized by the U1 snRNA-protein particle (snRNP), while the branch site is recognized by the U2 snRNP complexed with proteins at the 3′ splice site. In cancer cells, MYC upregulates the transcription of splicing components, SR proteins, driving aberrant splicing.

Alternative splicing of the RNA enables the regulation and generation of multiple mRNA and protein products from a single gene. During the alternative splicing, specific exons of a gene may be included within the final processed messenger RNA (mRNA) produced from that gene. The production of alternatively spliced mRNAs is regulated by a system of trans-acting proteins that bind to cis-acting sites on the primary transcript. These proteins include splicing activators that promote the usage of a particular splice site and splicing repressors that reduce the usage of a particular site. Mechanisms of alternative splicing exhibit high variability, and new examples are continually being discovered, particularly through the utilization of high-throughput techniques. Abnormal variations in splicing are also implicated in diseases, with a significant proportion of human genetic disorders resulting from splicing variants [27,28] (Figure 1).

## 3. Recurrence of Mutations in Splicing Factors in Hematological Malignancies and Their Effects on Splicing

**Figure 2:**
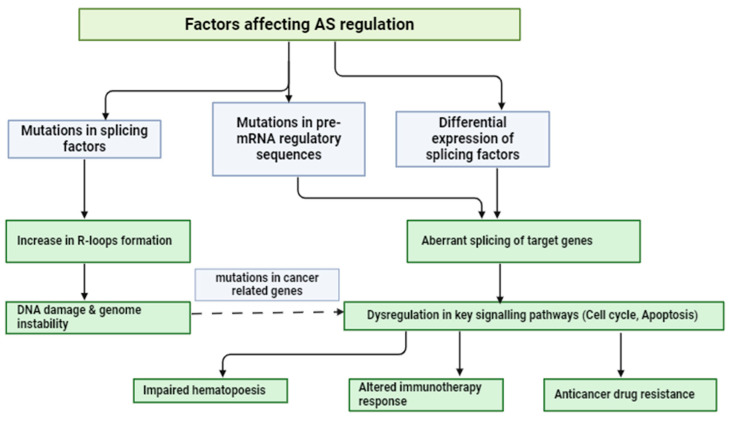
The pathophysiology of hematologic malignancies is influenced by aberrant regulation of alternative splicing (AS). Transcription disruption may be caused by early spliceosome mutations that impact pre-spliceosome assembly, hence promoting R-loop formation. However, because rapidly dividing cells exhibit relatively high sensitivity for R-loop-related DNA damage—likely because they lack the opportunity for DNA repair—cell division kinetics may be particularly relevant to the outcomes of DNA/RNA hybrid formation. Consequently, genomic instability brought on by inadequate repair of damaged DNA might accumulate a mutation burden. However, certain hematological splicing-factor-mutated cancers do not show signs of genomic instability; hence, more research is needed to determine the precise mechanism underlying transcription disruption, extensive R-loop creation caused by specific spliceosome-related mutations, and their interaction with AS events.

While the mechanics of alternative splicing and constitutive splicing are similar, several factors influence the splice site selection process. It has been found that hematological malignancies are more likely to acquire medication resistance when aberrantly spliced isoforms are produced. There is growing evidence that suggests AS abnormalities may have a role in the leukemic transition, the progression of cancer, and the response to treatment (Figure 2). 

Mutations in genes encoding splicing factors are among the most frequently observed genetic abnormalities in patients with various forms of myeloid neoplasms and several types of lymphoproliferative disorders, as well as in individuals with clonal hematopoiesis. These mutations implicate aberrant RNA splicing. 

The protein and RNA components of the splicing machinery are susceptible to mutations at highly precise residues, and many of these mutations modify splicing in ways different from loss of function. Mutations at highly specific residues enable the generation of mRNA with new aberrant sequences, some of which may play a crucial role in the pathogenesis of different disorders within the disease. These aberrant sequences could also represent novel targets for therapy. Research has demonstrated that cells with important splicing-factor mutations or changes are more susceptible to additional genetic or chemical perturbations. Using this information, a number of early-phase clinical trials are presently exploring these findings [21,29,30,31].

### 3.1. U2AF1 Mutations

U2AF1 constitutes an integral component of the spliceosome, and alterations within the U2AF1 gene represent prevalent genetic aberrations observed across various myeloid neoplasms and several forms of lymphoproliferative disorders (Table 1) [32]. These mutations can significantly compromise hematopoiesis, influence tumor progression, adversely impact disease prognosis, and facilitate leukemic transformation. U2AF1 plays a crucial role in mRNA splicing by recognizing the intronic 3′ splice site and contributes to the assembly of the U2AF complex as a heterodimer alongside U2AF2. Within the two zinc fingers of U2AF1, heterozygous hotspot mutations at S34 or Q157 result in sequence-specific aberrant splicing patterns that suggest possible changes in RNA binding affinity [33,34]. It is as yet unknown how these mutations affect the interactions between U2AF1 and U2AF2-RNA. Nonetheless, creating novel therapeutic strategies requires a thorough grasp of the link between structure and function. These tactics could focus on correcting RNA binding and splicing anomalies or taking advantage of them [33,35]. Mutation of the U2AF1 gene occurs in approximately 5% to 10% of the patients diagnosed with MDS [11]. 

### 3.2. SF3B1 Mutations

Numerous patients with diverse myeloid neoplasms and lymphoproliferative diseases frequently have mutations in the SF3B1 gene [21,48]. SF3B1 is the main component of the U2 small nuclear ribonucleoprotein (U2 snRNP), which is primarily responsible for recognizing the 3′ splice site at intron–exon junctions [20,48].

It is as yet unclear how these mutations, which affect the two zinc fingers of U2AF1, affect SF3B1-U2AF2-RNA interactions [48]. In myeloid malignancies, mutations in SF3B1 are most prevalent in myelodysplastic syndromes (MDS). However, recent data indicate that mutations in SF3B1 are also observed in AML, MPN, and the overlapped syndrome MDS/MPN [12,49,50]. Many studies have demonstrated that mutations in SF3B1 lead to diverse alterations in splicing, affecting numerous genes. However, these alterations converge on commonly dysregulated pathways and cellular processes, primarily centered around RNA splicing, protein synthesis, and mitochondrial dysfunction. This suggests shared mechanisms of action in MDS. It seems that SF3B1 mutations cause a decrease in mitochondrial respiration, which causes cells to increase glycolysis to make up for deficiencies in mitochondrial metabolism. As a result, cells become increasingly vulnerable to suppression of glycolysis [51]. 

In chronic lymphocytic leukemia (CLL), mutations in SF3B1 are observed in approximately 5% to 15% of diagnosed patients. These mutations are associated with a shorter time to initial treatment and reduced overall survival [36,37]. Mutation of SF3B1 is linked to inferior survival outcomes in both treatment arms, but it has been demonstrated not to be associated with progression-free survival in multivariant analysis [52]. Mutations in SF3B1 have been found to alter branch-point usage and 3′ splicing site selections. Recent studies, including those of the Manley group, suggest that SF3B1 mutations result in aberrant splicing by disrupting the connection of the SF3B1 with spliceosome [53,54]. 

### 3.3. SRSF2 Mutations

SRSF2, a member of the serine/arginine-rich (SR) protein family, plays a significant role in both constitutive and alternative splicing processes. It serves as a pivotal factor in promoting exon inclusion by binding to an exonic splicing enhancer sequence (ESE) [38,55,56,57]. As a member of the splicing family, SRSF2 is crucial for healthy hematopoiesis [58]. Patients with myelodysplastic syndrome (MDS) have been found to contain SRSF2 mutations, particularly the p95 mutation, which is associated with lower survival rates in MDS patients [39,40,41,59].

According to recent genome-wide sequencing research, MDS is linked to mutations in the genes that encode splicing factors [58]. The mutations in SRSF2 guide oncogenesis by activating a global program of aberrant alternative splicing in hematopoietic cells [60,61]. SRSF2 mutations are observed in approximately 5% to 15% of all MDS cases, but their prevalence is higher in chronic myelomonocytic leukemia (CMML) [11,42,62]. The mutation of the SRSF2 in primary myelofibrosis is found in 17% of cases [43]. Mutations of SRSF2 are more frequently observed in secondary acute myeloid leukemia (AML) derived from MDS or MPN [63]. The frequency of SRSF2 mutation in AML patients is approximately 10%, whereas in myelodysplastic syndrome (MDS) patients it ranges from 20% to 30%, and it is approximately 50% in patients with CMML [11,62,64].

### 3.4. ZRSR2 Mutations

One splicing factor that is essential to the control of the RNA splicing process is ZRSR2 (zinc finger-, CCCH domain-, and RNA-binding motif-containing serine/arginine-rich protein 2) [65]. 

Due to the aberrant splicing of LZTR1 (leucine zipper-like transcriptional regulator 1), mutations in the splicing factor ZRSR2 have been associated with a number of illnesses, including myelodysplastic syndromes (MDS) and predispositions to cancer [65]. ZRSR2 mutations are linked to a process known as minor intron retention, which is responsible for several cancer predispositions and MDS [44]. 

The generation of interferon, apoptosis, and compromised dendritic cell inflammatory signaling is linked to ZRSR2 mutations. This reveals a tumor suppressor mechanism connected to both sex and lineage [44].

Approximately 5% of patients diagnosed with MDS have been shown to be associated with ZRSR2 mutations [11,45]. These mutations have a higher risk of associating with AML transformations [66]. ZRSR2 has the ability to recognize and recurrently mutate through minor spliceosome [46]. The role of ZRSR2 in the splicing machinery is not yet fully understood. ZRSR2 is an RNA-binding protein (RBP) believed to interact with the 3′ splicing site of U12-type introns. Data from Madan et al. demonstrate that mutations in ZRSR2 primarily alter the splicing of U12-type introns by causing minor intron retention [46].

### 3.5. snRNA Mutations

Genetic anomalies in patients with various forms of lymphoproliferative diseases and myeloid neoplasms have been linked to mutations in genes encoding RNA splicing factors [20]. 

Small nuclear RNAs (snRNAs) U1 and U11 are a class of RNA molecules involved in RNA splicing. They are responsible for recognizing the 5′ splicing site in minor and major spliceosomes [67,68]. It has been demonstrated that aberrant splicing caused by mutations in snRNAs contributes to a number of illnesses, including cancer [47]. Shuai et al. identified mutation of U1 and U11 snRNA in patients diagnosed with CLL, diffuse large B-cell lymphoma, and sonic hedgehog in other types of cancers as well. These mutations cluster at the third base in U1 and U11 snRNAs, which base pairs to the 5′ splice site. Logically, the RNA sequencing of CLL cells bearing U1 snRNA mutations has identified changes in 5′ splicing site usage [67,68]. 

### 3.6. HNRNPH1 Mutations

Pertaining to the hnRNP (heterogeneous nuclear ribonucleoproteins) family, HNRNPH1 (heterogeneous nuclear ribonucleoprotein H1) is a protein that functions in RNA binding, among other processes [69]. 

According to a recent study, patients with chronic myeloid leukemia (CML) had greater levels of HNRNPH1, and this upregulation is linked to the disease’s progression [69]. A study, through the knockdown of HNRNPH1, demonstrated inhibited cell proliferation and promoted cell apoptosis in CML cells. Significantly, HNRNPH1 knockdown increased imatinib sensitivity in CML cells [69]. According to a different study, splicing of HNRNPH1 is impacted by silent and non-coding mutations, which also negatively impacts the prognosis of mantle cell lymphoma patients [70]. Mutations in HNRNPH1 occur around or within the single exon and serve to promote an isoform of the HNRNPH1 that escapes NMD. The association of mantle cell lymphoma (MCL) with HNRNPH1 mutations leads to increased expression of both the protein and HNRNPH1 mRNA [71]. Clinically, mutations in HNRNPH1 are associated with poor outcomes in MCL [72]. 

## 4. Mutations in Additional Alternative Splicing Components

### 4.1. BCAS2 (Breast Cancer Amplified Sequence) Mutations in Hematopoiesis

During zebrafish development, the gene BCAS2 is essential for maintaining the hematopoietic stem and progenitor cell (HSPC) population (Table 2) [73]. Hematopoietic stem and progenitor cells (HSPCs) and their derivatives during definitive hematopoiesis were found to be severely impaired in the work carried out on zebrafish with BCAS2 deletion [73]. The study also showed that BCAS2 deletion causes aberrant HSPC apoptosis by inducing alternative splicing of Mdm4 and elevation of p53 activation during HSPC development [73].

### 4.2. PRMT5 (Protein Arginine Methyltransferase 5) Mutations in Hematopoiesis

A protein called PRMT5 is essential to hematopoiesis, which is the process by which new blood cells are formed [74]. According to research conducted by Hamard et al., PRMT5 is a master regulator of erythropoiesis and is essential to the production of red blood cells. When fetal liver cells lack PRMT5, the outcome is embryonic mortality because the genes that encode DNA methyltransferase 3A and 3B are overexpressed in PRMT5-null embryos, causing severe anemia (Table 3) [74]. According to this study, PRMT5 inhibits erythropoiesis through a variety of methods, one of which is the regulation of DNA methyltransferase 3A protein levels [74]. According to a different study by Sapir et al., PRMT5 is inversely linked with patient survival and overexpressed in a variety of cancer types [76]. 

### 4.3. DDX41 (DEAD-Box Helicase 41) Mutations in Hematopoiesis

According to research, the DDX41 gene, which encodes the DEAD-Box Helicase 41 RNA helicase, is essential for the process of hematopoiesis, or the creation of new blood cells [75]. 

It is estimated that between two and five per cent of patients with AML and MDS have DDX41 mutations. A high frequency of normal karyotypes, a propensity toward cytopenia in the bone marrow and peripheral blood, a relatively good prognosis, and a late age of onset are some of the unique characteristics that define this disease subtype [75,77]. 

The concentration of DDX41 is primarily in the nucleus of the cell, where it carries out its functions as an RNA helicase. However, the expression of DDX41 has also been found in the cytoplasm of cells. It participates in the interferon I production pathway by recognizing foreign cytoplasmic DNA. Upon recognition, it signals the STING (stimulator of interferon genes) pathway in the cytoplasm. In the nucleus, DDX41 is believed to regulate the transcriptional elongation process signaling RNA polymerase II (Pol II) to slow down the elongation while the splicing process is taking place [78,79]. 

Myelodysplastic syndromes (MDS) and acute myeloid leukemia (AML) are indeed associated with disrupted splicing mechanisms. Research indicates that mutations in splicing-factor genes are prevalent in various myeloid neoplasms, including MDS and AML, with over 50% of MDS patients carrying such mutations. These mutations often lead to aberrant RNA splicing, which plays a critical role in the pathogenesis of these diseases [75,80].

### 4.4. Therapeutic Implications of Alternative Splicing Factor Mutations 

Cells can perform complicated biological tasks and diversify their proteome through a mechanism called alternative splicing [81]. The genesis and progression of cancer are fueled by aberrant splicing, which can be caused by aberrant spliceosomes or by distinct splicing factors [81]. 

**Table 4:** Therapeutic implications of alternative splicing-factor mutations across various hematological malignancies.

Splicing Factor	Mutation Hotspot	Associated Disease	Mechanism of Action	Clinical Significance	Reference
U2AF1	S34, Q157	Myeloid neoplasms, various lymphoproliferative disorders	Alters RNA binding affinity, affects 3′ splice site recognition	Compromises hematopoiesis, impacts disease prognosis, facilitates leukemic transformation	[82,83,84,85,86]
SF3B1	K700E, H662Q	Myelodysplastic syndromes (MDS), chronic lymphocytic leukemia (CLL)	Affects 3′ splice site recognition reduces mitochondrial respiration	Associated with shorter time to treatment, reduces overall survival	[87,88,89]
SRSF2	P95	Myelodysplastic syndromes (MDS), chronic myelomonocytic leukemia (CMML)	Promotes exon inclusion by binding to exonic splicing enhancers	Linked to poor survival in MDS, prevalent in secondary AML	[60]
SRSF2	P95	Myelodysplastic syndromes (MDS), chronic myelomonocytic leukemia (CMML), secondary acute myeloid leukemia (AML)	Promotes exon inclusion by binding to exonic splicing enhancers	Linked to poor survival in MDS, prevalent in secondary AML	[63,90]
ZRSR2	Various	Myelodysplastic syndromes (MDS)	Leads to minor intron retention	Higher risk of AML transformation, impaired inflammatory signaling	[46,91,92]
snRNAs	Various	Myeloid neoplasms, lymphoproliferative disorders, CLL, diffuse large B-cell lymphoma, sonic hedgehog tumors	Cause aberrant RNA splicing	Associated with production of aberrant mRNA transcripts and contribute to various diseases	[68,93,94]
HNRNPH1	Various	Chronic myeloid leukemia (CML), mantle cell lymphoma (MCL)	Affects RNA binding, splicing regulation	Upregulated in CML associated with disease progression and poor outcomes in MCL	[45]

Targeting alternative splicing factors as a novel avenue for cancer treatment was covered in a recent review article written by a variety of authors [81]. Murphy et al. give a summary of the alternative splicing factors involved in aberrant splicing during processing in various cancer cells, emphasizing serine/arginine-rich (SR) proteins and their newly discovered roles in the onset and spread of cancer [81]. 

The authors also discuss the new mapping of the spliceosome, the splicing factors that are linked to it, and how these factors relate to cancer. Because of this finding, new therapeutic strategies that take advantage of alternative splicing’s broad influence are now possible [81]. The article’s discussion of recently discovered small-molecule spliceosome inhibitors ushers in a new age of cancer treatment [81]. 

Various small molecules are being tested to determine their ability to bind to different alternative splicing factors and explore their potential for therapy in cancer treatment (Table 4). Many of these small molecules are currently undergoing preclinical studies. For example, pladienolides A-G, herboxidiene (GEX1A), Fr901463, FR901464, FR9011465, meayamycin B, and spliceostatin A have demonstrated the ability to bind with splicing factor SF3B1 and destabilize snRNP U2, leading to cell cycle arrest and disruption of spliceosome assembly. In CLL, resistance to fludarabine has been observed. Additionally, splicing variants of the FPGS (folylpolyglutamate synthetase) gene have been implicated in drug resistance in T-cell acute lymphoblastic leukemia and acute lymphoblastic leukemia against methotrexate, dexamethasone, mitoxantrone, and prednisolone. Similarly, in acute myeloid leukemia, certain splicing variants have been associated with drug resistance against cytarabine and doxorubicin (reviewed by Temaj et al., 2023) [23]. Resistance to fludarabine in chronic lymphocytic leukemia (CLL) is primarily associated with several biological mechanisms, particularly involving dysregulated splicing and mutations in splicing factors [95]. 

### 4.5. The Global Evidence of Splicing Dysregulation in Cancer

There is significant evidence of global splicing dysregulation in cancer, characterized by alterations in RNA splicing mechanisms that contribute to tumorigenesis. This dysregulation occurs regardless of whether mutations in splicing factors are present. Alterations in splicing factors, both through mutations and changes in expression levels, play a crucial role in the misprocessing of pre-mRNA into mature mRNA, leading to the production of oncogenic splicing variants. These variants can enhance malignancy by promoting cell proliferation, evading apoptosis, and facilitating metastasis and angiogenesis. Research has demonstrated that a wide array of cancers exhibit unique splicing signatures, with studies revealing thousands of cancer-specific splice variants across different tumor types. For instance, specific splicing variants of genes like BCL-x and MCL1 have been linked to apoptosis regulation, while variants in HER2 and BRCA1 are associated with poor prognosis in breast cancer. In multiple myeloma, around 40% of splicing factors have been found to be dysregulated, correlating with adverse clinical outcomes. Additionally, hepatocellular carcinoma studies have identified distinct RNA splicing signatures associated with tumorigenesis, implicating genes such as KRAS and BRCA1 in altered splicing events. Overall, the evidence shows that global splicing dysregulation is a hallmark of cancer, driven by both mutations and non-mutational changes in splicing factors. Understanding these mechanisms provides valuable insights into cancer biology and opens avenues for targeted therapeutic strategies aimed at correcting or inhibiting aberrant splicing events. Further research is essential to fully elucidate the complexities of this dysregulation and its implications for cancer treatment [96,97,98,99,100]. 

### 4.6. Germline Syndromes Associated with Dysregulated Splicing

Germline syndromes associated with dysregulated splicing can predispose individuals to various cancers, particularly hematological malignancies. Notably, several syndomes have been identified where mutations in splicing factors or related genes contribute to an increased risk of developing malignancies such as MDS (myelodysplastic syndromes), acute myeloid leukemia (AML), familial syndrome (FM), and other genetic disorders such as Fanconi anemia (FA) and Li–Fraumeni syndrome [101,102]. 

### 4.7. Clinical Studies in the Implication of Splicing Factors in Hematological Malignancies

Recent clinical studies have focused on the implications of splicing-factor mutations in hematological malignancies, particularly acute myeloid leukemia (AML), myelodysplastic syndromes (MDS), and chronic myelomonocytic leukemia (CMML). These studies explore the potential for targeted therapies that address the unique molecular alterations associated with these mutations. The phase II clinical trial (NCT05024994) investigated the anti-cancer effect of the sulfonamide E7820 in patients with relapsed/refractory (R/R) splicing- factor-mutant AML, MDS, or CMML. The trial aimed to assess the drug’s efficacy in degrading RBM39, a splicing factor critical to the survival of AML cells. Although the study was terminated early due to limited clinical efficacy—no patients meeting primary endpoints—the results indicated a modest decrease in splicing factor mutation allele frequency and significant changes in alternative splicing patterns induced by E7820 treatment [103]. Research has shown that approximately 60% of patients with MDS exhibit mutations in spliceosome genes. These mutations are believed to contribute to oncogenic processes by altering pre-mRNA splicing. Clinical trials targeting these mutations are ongoing, focusing on pharmacological approaches that leverage the dependency of mutant cells on aberrant splicing mechanisms [104,105].

## 5. Conclusions

Alternative splicing is a complex transcriptional regulatory mechanism that affects nearly 95% of all protein-coding genes and occurs in nearly all human organs. In addition to causing aging, infection, inflammation, immune system and metabolic problems, and other conditions, aberrant alternative splicing has been linked to a number of neurological illnesses and cancers [106]. 

In the future, resistance to anticancer drugs will likely become a central topic in cancer biology. Many factors contribute to cellular behavior, and data from various research groups indicate that alternative splicing plays a pivotal role in various cancer types. Alternative splicing can impact resistance to anticancer drugs by altering drug targets, influencing drug metabolism, enabling cancer cells to bypass drug targets, activating survival pathways, and dysregulating splicing factors. Understanding this intricate relationship is crucial for the development of effective cancer therapies.

Novel approaches to therapy, such as alternative splicing, have been put forth to target exon junctions in mutant mRNA to obstruct protein coding or to stop the expression of disease-associated proteins [107]. Antisense oligonucleotides (ASOs) that selectively change splicing processes in order to rectify abnormal mRNA processing have also been suggested as a potential therapeutic alternative [108]. Phprotein-codingarmacological therapy based on the targeting of alternative splicing factors represents a promising avenue for cancer therapy, offering innovative approaches to combat the complexities of cancer progression and treatment resistance.

## Figures and Tables

**Table 1:** Summary of the alternative splicing factors and their associated disease.

Splicing Factor	Mutation Hotspot	Associated Disease	Mechanism of Action	Clinical Significance	Reference
U2AF1	S34, Q157	Myeloid neoplasms, various lymphoproliferative disorders	Alters RNA binding affinity, affects 3′ splice site recognition	Compromises hematopoiesis, impacts disease prognosis, facilitates leukemic transformation	[31,32,33]
SF3B1	K700E, H662Q	Myelodysplastic syndromes (MDS), chronic lymphocytic leukemia (CLL)	Affects 3′ splice site recognition, reduces mitochondrial respiration	Associated with shorter time to treatment, reduced overall survival	[20,35,36,37]
SRSF2	P95	Myelodysplastic syndromes (MDS), chronic myelomonocytic leukemia (CMML), secondary acute myeloid leukemia (AML)	Promotes exon inclusion by binding to exonic splicing enhancers	Linked to poor survival in MDS, prevalent in secondary AML	[10,38,39,40,41]
ZRSR2	Various	Myelodysplastic syndromes (MDS)	Leads to minor intron retention	Higher risk of AML transformation, impaired inflammatory signaling	[42,43]
snRNAs	Various	Myeloid neoplasms, lymphoproliferative disorders, CLL, diffuse large B-cell lymphoma, and sonic hedgehog	Cause aberrant RNA splicing	Associated with the production of aberrant mRNA transcripts and contribute to various diseases	[44,45]
HNRNPH1	Various	Chronic myeloid leukemia (CML), mantle cell lymphoma (MCL)	Affects RNA binding, splicing regulation	Upregulated in CML, associated with disease progression and poor outcomes in MCL	[46,47]

**Table 2:** Summary of the alternative splicing factors and their role in normal hematopoiesis.

Splicing Factor	Description	Reference
SF3B1	The SF3B1 is the main component of the U2 small nuclear ribonucleoprotein (U2 snRNP), whose main function is to recognize the 3′ splice site at intron–exon junctions	[11]
U2AF1	The U2AF1 is part of the spliceosome, and mutations of the U2F1 are among the most common genetic abnormalities in patients with all types of myeloid neoplasms and several types of lymphoproliferative disorders	[32]
SRSF2	The SRSF2 is a member of the serine/arginine-rich (SR) family, which is shown to give a higher contribution in the constitutive and alternative splicing process and play the pivotal role in the promotion of exon by connecting to the exonic splicing enhancer sequence (ESE)	[38,55,56,57]
ZRSR2	The ZRSR2 is a splicing factor that is shown to play a crucial role in the regulation of the RNA splicing process	[65]
BCAS2 (breast carcinoma amplified sequences)	The gene BCAS2 plays a pivotal role in hematopoietic stem and progenitor cell (HSPC) maintenance during zebrafish embryogenesis	[73]
PRMT5 (protein arginine methyltransferase 5)	The PRMT5 is a protein that plays a crucial role in hematopoiesis, during the process of blood cell formation	[74]
DDX41 (DEAD-box Helicase 41)	The DDX41 is a DEAD-box type RNA helicase; it has been shown that DDX41 plays a crucial role in hematopoiesis	[75]

**Table 3:** Summary of the gene related to alternative splicing and hematopoiesis.

Gene	Mutation Type	Function	Clinical Significance	Reference
BCAS2	Mutations in BCAS2 trigger alternative splicing of Mdm4 and upregulation of p53 activation during HSPC development, leading to abnormal apoptosis of HSPCs.	Plays a pivotal role in the maintenance of hematopoietic stem and progenitor cell (HSPC) maintenance during zebrafish embryogenesis.	Associated with severe impairment of HSPCs and their derivatives during definitive hematopoiesis in zebrafish, potentially contributing to hematopoietic disorders.	[69]
PRMT5	Deletion of PRMT5 results in severe anemia and increased expression of DNA methyltransferase 3A and 3B proteins, negatively regulating erythropoiesis.	Plays a crucial role in hematopoiesis and acts as a master regulator of erythropoiesis.	Implicated in embryonic lethality, severe anemia, and aberrant DNA methylation patterns, potentially contributing to hematopoietic disorders and anemia.	[70,71]
DDX41	Mutations in DDX41 are found in approximately 2-5% of AML and MDS patients, associated with late onset, cytopenia, and favorable prognosis.	Participates in the regulation of transcriptional elongation and interferon I production pathways, primarily localized in the nucleus and involved in splicing regulation.	Associated with distinctive phenotypes in AML and MDS patients, including late onset, cytopenia, and favorable prognosis, potentially affecting disease progression.	[72,73,74,75,76]

## Data Availability

Not applicable.
